# Supplementary material for: Are carbon emissions trading and green financial instruments synergistic? -Comprehensive quantitative research based on content analysis
Source: PLoS One. 2024 Mar 7;19(3):e0298601. doi: 10.1371/journal.pone.0298601 (PMC10919601; doi:10.1371/journal.pone.0298601)
Supplement: S1 Data — (ZIP) [file pone.0298601.s001.zip › Data/地级市Z_F工作报告.zip!/╣π╬≈/░┘╔1⁄2╩╨/└┤╫╘╣1⁄2╓┌║┼í╛┬φ┐╦╩2╛▌═°í┐_2016.docx]

一、过去五年工作回顾

过去五年，是百色发展极不平凡的五年。面对严峻复杂的形势和多重困难的挑战，在自治区党委、政府和市委的正确领导下，我们紧紧依靠和团结全市各族人民，以坚定的信心和巨大的勇气，开拓进取，攻坚克难，胜利完成市第三届人民代表大会第一次会议确定的各项目标任务，改革开放和现代化建设取得了新的重大成就，为决战贫困决胜小康打下坚实基础。

——综合实力大跨越。地区生产总值达980.4亿元、逼近1000亿元，是2010年的1.4倍，居全区第6位。人均GDP达3585美元，进入中等收入地区行列。固定资产投资突破1000亿元，达1022亿元，是2010年的1.68倍。规模以上工业总产值突破1000亿元，达1285亿元，是2010年的2.1倍。财政收入突破100亿元，达114.5亿元，是2010年的1.6倍。全市12个县（市、区）财政收入进入亿元行列，平果、靖西、田东、田阳、德保、右江区财政收入超10亿元，平果率先突破20亿元大关。

——经济韧性大增强。三次产业结构由2010年的18.3:54.7:27调整为17.3:52.2:30.5，服务业占比提高3.5个百分点，产业结构向更优形态演进。对外贸易进出口总额翻两番。社会消费品零售总额突破200亿元，达221亿元，是2010年的1.94倍。金融机构存贷款余额突破1000亿元，达1660亿元，是2010年的1.84倍，抵御风险能力增强。园区承载能力提高，100亿元以上工业园区达到5家。一批新经济龙头企业落户百色，带动形成百色新产业、新业态、新商业模式。

——发展条件大改善。9个县（市、区）通高速公路，县县通二级路，所有乡镇通客车，基本实现村村通水泥路。形成铁路、高速路、航空、水运、口岸等综合立体交通枢纽，大通道、大平台、大通关建设实现新突破，通边达海、连接南北的骨干网基本形成，对内对外开放的环境日臻完善。

——民生福祉大提升。累计新增城镇就业10.9万人、农村劳动力转移就业30.8万人。累计减贫101.3万人。城镇居民人均可支配收入年均增长9.3%。农民人均纯收入年均增长14.3%，连续5年保持两位数增长，增速高于全国全区平均水平，群众获得感普遍提高。

五年来，我们主要抓好了以下工作：

（一）争取进入中央自治区顶层设计，战略地位重要性进一步巩固。争取到国家批复以百色为核心区的《左右江革命老区振兴规划》，百色发展上升为国家战略。生态型铝产业示范基地获得国家和自治区批复。成为全国首个政策性金融扶贫实验示范区。被自治区确定为“广西开放发展新门户”。国家《珠江—西江经济带发展规划》《滇桂黔石漠化片区区域发展与扶贫攻坚规划》《全国资源型城市可持续发展规划》覆盖百色。

（二）突破铝工业发展瓶颈，“再造一个工业百色”取得重大进展。全面推进生态型铝产业示范基地建设，区域电网一期工程建成运营，铝工业用电价格大幅下降。陆续建成5个煤电铝一体化项目。铝材向精深加工发展，产量达214万吨。高新技术产业增加值比重不断提升。碳酸钙非金属新材料等新兴产业布局加快。规模以上工业增加值411亿元，是2010年的1.9倍。

（三）加快培育新商业模式，现代服务业蓬勃发展。引进交通银行、柳州银行、北部湾银行等一批新的金融机构，金融业增加值36.1亿元，比“十一五”翻一番，占地区生产总值3.68%。建成淘宝特色中国百色馆、京东商城百色馆、阿里旅游百色旗舰店等5大电商平台。亿丰国际商博城、恒宁城市广场等一批城市综合体加快推进。中心城区“十大市场”、壮乡河谷、古鼎香等交易市场建成。汽车销售“一站式”服务平台运营。开通了百色至北京果蔬绿色专列。房地产业健康发展。百色起义纪念园等一批景区创建国家5A、4A级景区取得新进展。特色旅游名县创建工作稳步推进。南宁—靖西旅游专线开通。全市旅游接待人数增长1.4倍，旅游总收入增长2.5倍。

（四）稳步发展特色农业，品质品牌提质增效。推进百万亩芒果、百万亩油茶、林产“五个百万亩”工程建设。芒果面积达92万亩，跃升为全国最大产区。蔬菜面积达182万亩。油茶面积达150万亩。中草药总面积突破30万亩。桑园总面积32.6万亩。火龙果、柑桔、香蕉、猕猴桃、茶叶等面积稳步扩大。林下养鸡出栏量增幅居全区第1位。渔业产量增幅连续五年保持全区第1位。市级以上重点龙头企业达99家。建成11个农产品质量安全追溯平台，“三品”基地认定面积突破200万亩。田阳、凌云成功创建自治区级现代特色农业示范区。乐业、西林成功创建全国有机农业示范基地。全市累计有18个农产品先后获得国家农产品地理标志、原产地保护产品认证、地理标志证明商标。

（五）全面提升交通互联水平，门户城市地位凸显。高速铁路百色段、德靖铁路建成通车，新增铁路298公里，总里程达568公里。新建公路2800公里，总里程达1.66万公里。建成百色至隆林、百色至靖西、靖西至那坡、平果至马山4条高速公路413公里，总里程达598公里。实现乡乡通沥青（水泥）路，村村通公路。开通百色至上海、广州、深圳、天津、重庆、桂林等6条航线。右江千吨级黄金水道基本建成。

（六）加快推进新型城镇化，“百色新城”快速崛起。常住人口城镇化率达34.1%。全市建成区面积136.3平方公里，增加26.9平方公里。中心城区主骨架形成。百东新区建设快速推进。右江田阳一体化加快形成。平果成为国家新型城镇化综合试点县。靖西撤县设市。百色园博园、半岛公园、沙滩公园，百色高中新校区、体育中心等一批中心城区项目投入使用，田州古城、靖西绣球城、平果大学城、田东湿地公园等一批县域城镇项目陆续建成。“国家森林城市”创建顺利推进。百色建成“国家园林城市”、“国家卫生城市”、“广西森林城市”，成为全区首个县县成功创建“广西卫生县城”、“广西园林城市”的地级市。

（七）金融创新顺势破题，改革红利持续迸发。农村金融改革走在全国前列。创建全国第一个政策性金融扶贫实验示范区，设立左右江老区振兴发展基金、百色城镇化发展基金、百色扶贫开发基金，筹建左右江投资开发银行、百色银行，沿边金融综合改革成效初显，金融“洼地”加快形成。区域电网效果显著，电价“洼地”初步显现。百投集团、百矿集团、福地金融集团、百东投资公司等国有企业集团发展壮大。城市管理综合执法体制改革、司法体制改革试点工作启动实施。农村综合改革、医药卫生体制改革扎实推进。百色国家农业科技园区“园镇一体化”改革稳步实施。集体林权制度改革走在全区前列。简政放权进一步扩大。商事制度改革全面实施。非公经济快速发展。“十二五”末，全市个体工商户户数、企业户数分别是2010年的1.2倍和2.7倍。

（八）夯实跨境跨省平台，开放合作领域拓展。中越龙邦—茶岭跨境经济合作区、龙邦万生隆国际商贸物流中心、中国—东盟农产品交易中心建设取得实质性突破。开通了与越南高平省的国际客货运输通道和跨国旅游线路。中国—东盟现代农业展示交易会成为中国—东盟博览会暨商务与投资峰会的重要组成部分。平孟口岸升格为国家一类口岸，龙邦口岸成功申报粮食、水果进境指定口岸。全市6家金融机构从事跨境人民币结算业务总量达54亿元，覆盖19个国家和地区。外贸进出口总额翻了两番。与广州对口协作成为东西部合作典范。与云南文山州、贵州黔西南州启动建设跨省区经济合作园区，与河池共建巴马长寿养生国际旅游区，与钦州开展原油委托加工。打通了香港、深圳、上海等地项目和资金进入百色的新渠道，招商引资到位资金累计1957亿元，是“十一五”的2.6倍。

（九）扎实推进“美丽百色”建设，生态环境不断改善。森林覆盖率达67.37%。森林面积、植树造林面积、森林活立木蓄积量三项指标居全区第一。水土流失治理380平方公里，石漠化综合治理61.5万亩，户用沼气池建设44.5万座，村屯绿化完成6819个。创建区、市生态村252个。田东、凌云、乐业、西林成为全国生态文明示范工程试点县。乐业成为世界长寿之乡，凌云成为中国长寿之乡。“十二五”主要污染物减排任务全面完成，万元GDP能耗提前一年达标。全市村级生活垃圾收集转运处理基本实现全覆盖，城市生活垃圾无害化处理率达100%。空气质量优良率达96.7%。饮用水源地水质达标率达100%。

（十）实施“扶贫开发优先”，贫困人口大幅减少。实施“整村推进”、“十个到村到户”、兴边富民行动等扶贫攻坚工程，产业发展全面覆盖，全市投入产业扶贫项目财政资金9.2亿元，累计扶持1104个贫困村、18.6万户、80.4万人。社会帮扶成效显著。教育扶贫实现困难学生资助全覆盖，贫困家庭子女就读普通本科高校、高等和中等职业院校全部实现应补尽补。率先在全区启动精准扶贫。“十二五”末，全市贫困人口下降到68.2万人，脱贫141个村，贫困发生率由49.95%下降到20.25%。

（十一）提升公共服务水平，社会事业全面发展。新建、扩建中小学标准化学校331所，新增幼儿园61所。学前教育入园率稳居全区前列。九年义务教育巩固率达88.9%。百色学子考录清华、北大等重点高校人数连年成倍增长，五年累计向高校输送本科学生5.47万人。高等教育规模稳居全区前列。获自治区科学技术进步奖26项、自然科学奖3项，顺利通过全国科技进步考核。9个项目被列入国家级非物质文化遗产保护名录。一批文艺精品获全区“五个一工程”奖。广播电视实现“村村通”。博物馆、图书馆等公共服务场所免费向全民开放。百色市籍运动员荣获世界冠军6个、亚洲冠军3个、全国冠军68个。成功举办系列国际国内体育赛事。城乡体育基础设施不断完善，全民健身运动蓬勃开展。

（十二）进一步加大民生投入，群众获得感显著增强。民生支出达959.6亿元，是“十一五”的3倍，占财政总支出80%以上。基本形成覆盖城乡的社会保障、医疗服务体系。城乡低保、五保对象、双拥优抚对象和0至3公里边民实现应补尽补。城镇登记失业率控制在4%以内。新建农村饮水安全工程4775处，农村自来水普及率达75%。新增、恢复和改善灌溉农田76万亩。建成保障性住房和棚户区改造7.1万套，完成农村危房改造21.7万户。养老服务机构由852个发展到1187个。人口计生工作连续5年保持全区先进。全市医疗卫生业务用房从60.2万平方米增加到104.4万平方米，医疗卫生条件得到极大改善。新农合参合率达98.9%，报销比例大幅提高。全市32家县级公立医院、179个基层医疗卫生机构完成了公益性转型。城镇居民人均可支配收入达24958元，年均增长9.3%。农民人均纯收入达6766元，年均增长14.3%，连续5年保持两位数增长。

（十三）不断完善社会治理体系，平安百色建设扎实推进。“六五”普法全面完成。连续两次荣获“建设平安广西活动先进市”。食品药品监管体系实现市县乡村四级全覆盖。“质量兴市”成效明显。安全生产持续稳定好转。应急救援能力水平不断提高。社会稳定风险评估、农村治安防控、边防“六联”管控、边境安全管控、跨省接边区域综治维稳协作进一步加强。人民调解、煤矿安全生产“百色经验”、基层应急管理规范化建设等工作得到国家、自治区肯定。

民族团结进步创建活动深入开展，民族团结和睦，边疆巩固安宁。国防后备力量建设和双拥共建取得新成果，连续两届荣获“全国双拥模范城”称号。信访、宗教、侨务、保密、税务、人事人才、水库移民、消防、统计、供销、粮食、检验检疫、通信、海关、海事、气象、邮政、地震、地矿、测绘、农机各项工作取得新成绩。哲学社会科学、新闻出版、决策咨询、地方志、档案、法律援助、妇女儿童、老龄、残疾人等事业取得新进步。工会、共青团、妇联、红十字会、科协等群团组织在经济社会发展中发挥了重要作用。

强化政府自身建设。坚决落实中央“八项规定”和国务院“约法三章”，大力开展“四风”问题专项整治。认真执行市人大及其常委会的决议、决定，自觉接受人大、政协和社会监督，广泛听取各民主党派、工商联、无党派人士意见，人大代表议案、建议和政协委员提案办结率100%。稳步推进政府机构改革和职能转变。完善政府重大决策程序和机制，进一步规范政府权力运行。全面建立政府法律顾问制度。行政机关负责人出庭应诉制度得到有效执行。公共资源交易平台运行进一步规范，政务公开和政府信息公开更加透明。审计监督力度加大。绩效管理加强。廉政建设和反腐败斗争取得新成效。

二、今后五年工作的总体要求、奋斗目标和主要任务

今后五年政府工作总体要求是：高举中国特色社会主义伟大旗帜，以马克思列宁主义、毛泽东思想、邓小平理论、“三个代表”重要思想、科学发展观为指导，深入学习贯彻习近平总书记系列重要讲话精神，全面贯彻落实中央“五位一体”总体布局、“四个全面”战略布局、自治区“两个建成”战略决策和市第四次党代会战略部署，贯彻创新、协调、绿色、开放、共享发展理念，坚持发展第一要务，着力打造“三中心两区一市”，基本建成左右江革命老区核心城市，坚决夺取决战贫困决胜小康的全面胜利。

实现全面脱贫。到2020年，现行标准下68.2万农村贫困人口、754个贫困村、11个贫困县（市、区）全部脱贫出列，历史性地摘掉贫困的帽子。

与全国全区同步全面建成小康社会。经济持续较快健康发展，地区生产总值和城乡居民人均可支配收入年均增长10%，高于全国、全区水平，比2010年翻一番以上；工业增加值年均增长10.5%；财政收入年均增长7.5%；固定资产投资年均增长10%；进出口总额年均增长25%；社会消费品零售总额年均增长10%；常住人口城镇化率达到45%；基本公共服务均等化水平不断提高，城乡发展更协调；全面深化改革取得决定性成果，开放合作取得新突破；生态环境保持良好，节能减排取得显著成效；各项事业全面发展；社会和谐稳定，民族团结和睦，边疆安宁繁荣。

基本建成左右江革命老区核心城市。百色在左右江革命老区发展中的引领地位显著增强。

实现今后五年发展目标，重点要抓好以下工作。

（一）全力以赴，奋力打赢脱贫攻坚战。

大力实施基础设施扶贫到村到户。推进84个行政村道路硬化工程、2338个20户以上自然屯通屯级路、163个20户以下自然屯通电，解决43.8万农村人口饮水安全问题，完成5.12万户的危房改造。到2016年底，实现行政村通村道路硬化率100%。到2017年，全面解决用电难问题，基本实现行政村宽带信息网络全覆盖。到2019年，实现20户以上通屯道路硬化率80%。

千方百计促进贫困群众增收。实施产业扶贫，建设一批扶贫产业综合示范区，培育一批农业产业化扶贫龙头企业和农民专业合作组织，发展水果、蔬菜、桑蚕、油茶、茶叶、中草药、水产畜牧等特色产业。发展乡村旅游扶贫、边境贸易扶贫、电子商务扶贫。到2019年，实现产业精准脱贫28万人。引导贫困群众以土地、林权、财政扶持资金、扶贫小额信贷资金入股龙头企业、合作社，获得固定分红和利润分成。实施农民工培训创业行动，引导农村贫困劳动力有序向非农产业和城镇转移就业，转移就业脱贫10万人以上。加快实施教育扶贫工程。提高贫困人口医疗补助标准，推动低保制度与贫困户的有效衔接，落实国家生态补偿政策，实现减贫32万人。

大力实施扶贫移民搬迁工程。完善扶贫移民搬迁安置区规划，落实配套政策，筹措162.5亿元，建设157个扶贫移民搬迁集中安置区。实施梯度搬迁、分类搬迁，完成6.28万建档立卡户25.1万贫困人口的搬迁任务。

全面建成政策性金融扶贫实验示范区。加快组建市县两级扶贫投融资平台公司和融资担保机构。推进政策性金融扶贫制度创新、产品创新、管理创新。建立财政资金与政策性金融扶贫资金协同配合机制。推动设立易地扶贫移民搬迁、贫困地区基础设施建设、特色产业发展、教育扶贫、光伏扶贫、旅游扶贫等贷款品种。实行扶贫开发贷款整区域综合授信，加大信贷投放力度。推进政策性银行与商业银行、农村合作银行、保险、证券、基金等机构的金融扶贫合作，发挥政策性金融引领作用，创造可复制、可推广的“百色经验”。

完善扶贫工作机制。建立扶贫大数据平台，实行扶贫信息动态管理。建立健全脱贫退出、返贫纳入机制，深入实施扶贫“双承诺、双认定、双确认”工作制度，为第三方评估脱贫提供信息平台。健全扶贫协作机制、定点扶贫机制和社会力量参与机制，全面推进与深圳市的扶贫协作。

（二）稳增长保发展，奋力推进产业转型升级。

提升工业经济发展水平。全面推进铝产业“二次创业”，加快推进煤电铝一体化和区域电网二期工程，建成生态型铝产业示范基地、铝精深加工基地，到2020年实现铝工业总产值1100亿元以上。壮大石化产业规模，优化石化产业结构。加快锰、铜等矿产资源综合开发，建设百色生态型锰铜产业示范基地。推进建材、机械制造、农林产品加工等产业提质升级。推动中小微企业快速发展。推动工业园区向创新型园区转型发展，打造产值超千亿元产业1个，超300亿元产业6个，超300亿元园区5个，超百亿元企业5家。到2020年，力争工业总产值达2500亿元以上。

加快发展现代农业。提高农业综合生产能力，推进高标准农田建设，确保粮食安全。大力实施百万亩芒果、百万亩柑桔、百万亩桑蚕、百万亩油茶等特色农业产业发展工程。推进国家级“南种北供冬菜园”、现代特色农业示范区建设。加快林产加工园区建设，建设现代林业产业示范区。做大牛羊产业、做优林下养鸡、做强现代渔业，打造现代生态养殖基地。大力提升农产品质量，推进“三品一标”认证，建设农产品溯源物联网监管云平台。到2020年全市建成农产品质量安全示范区24个以上，标准化示范园（基地）36个以上，新增国家地理标志保护产品10个以上，创建自治区级和国家级农业标准化示范区5个以上。

加速发展现代服务业。推进长寿养生国际旅游区建设，加快百色起义纪念公园、乐业大石围天坑群、靖西大峡谷创建国家5A级旅游景区，推进红色旅游核心区建设。到2020年，实现县县创有A级景区，旅游营业收入达500亿元以上。加快电子商务产业园建设。推进百色龙邦国际物流商贸、中国—东盟农产品交易中心等重点服务业集聚区建设。推进农村金融改革和沿边金融综合改革试验区建设，设立左右江投资开发银行、百色银行，实现金融服务业新突破。到2020年，全市金融业增加值翻一番，占地区生产总值比例达5%。

培育发展新兴产业。推进“互联网+”、物联网、云计算等新兴产业和业态，培育发展新经济。大力发展总部经济。加快推进碳酸钙产业基地建设，打造新的百亿元产业。大力发展新医药产业，在种植、制造、物流、医疗、健康管理产业链上培育新业态，到2020年，总产值达10亿元以上。推动节能环保产业发展，到2020年，综合利用节能环保产业产值达10亿元以上。

（三）推进基础设施再升级，奋力增强发展支撑力。

完善提升立体交通体系。建成云桂高速铁路、南昆铁路二线。建设云南文山—那坡—靖西—防城沿边铁路。力争黄桶—百色、靖西—龙邦铁路开工建设。开展百色—柳州铁路、桂林—河池—百色铁路、百色—兴义高速铁路前期工作。完善高速公路网络，建成百色—乐业、百色—河池、靖西—龙邦、田林—西林—罗平高速公路，实现县县通高速公路。推进国省干线、跨省区干道提级改造建设。提高右江黄金水道通航能力。大力推进百色新机场、乐业、德保通用机场建设。

加强水利基础设施建设。实施百色水库灌区工程。加快推进重点水源、小型农田水利、“五小”水利、高效节水灌溉，完善水资源合理配置和高效利用体系。加快推进病险水库除险加固、中小河流治理、城市防洪及易涝区治理，加强防灾减灾能力建设。推进小流域水土保持综合治理、水生态文明建设、饮用水水源地保护、水资源保护与污染监控工程建设，形成水资源保护和河湖健康保障体系。

全面提高能源保障能力。实施农网升级改造和城网改造工程。完善区域电网建设，提高电网保障能力，切实降低区域用电价格。合理开发水电，稳妥发展火电，因地制宜发展生物质发电，有序推进风能、太阳能利用。加快推进与大网和邻省市（州）电网合作互联。加强煤炭资源开发与合作。进一步开展右江盆地油气勘探开发。实施县县通天然气工程。

加强信息网络建设。实现新一代移动通信网、下一代互联网、数字广播电视网“三网融合”。加快物联网、云计算、大数据等新一代信息技术应用。实施“物联网”和“互联网+”行动计划，推动跨境电子商务、远程医疗、网络文化、智能电网、金融信息、网络安全等信息服务业发展。

（四）统筹城乡协调发展，奋力构建大百色新格局。

优化“三带三区四中心”城镇体系。打造东西向城镇发展带、南北向城镇发展带和沿边城镇发展带。加快推进右江河谷城乡一体化示范区、西北民族生态特色城镇片区、南部沿边开放开发特色城镇片区建设，形成百色中心城区主中心，平果、靖西、隆林三个副中心。加快百东新区建设，推动田阳撤县设区、平果撤县设市。实施大县城战略。

全面提升城市规划管理水平。推进城市总体规划、土地利用总体规划和经济社会发展规划等“多规合一”，优化市、县、乡城镇化发展规划，实现中心城区和各县（市）城区控制性详细规划全覆盖。推进市县城市管理执法体制改革。加快智慧城市、数字城市建设，建成数字化城市管理平台。实施城市违法建设专项治理五年行动计划。发展城市绿化，创建宜居城市。

加强城镇基础设施建设。优先发展城镇公共交通，到2020年，全市建成区平均路网密度达每平方公里8 公里以上，建成区道路面积率达15%以上，公共交通分担率达到20%以上。加快城乡污水处理设施建设与提标改造，到2020年，全市新增48座建制镇污水处理厂，污水处理率均达95%以上。加强生活垃圾处理设施建设，实现镇镇建成垃圾转运站，生活垃圾回收利用率达35%以上。推进城市地下综合管廊和海绵城市建设试点。到2020年基本完成城镇棚户区、城中村和危房改造。

推进城乡协调发展。大力发展城镇经济，培育建设20个以上经济强镇和一批特色小镇。实施易地扶贫搬迁工程，推动25万左右的贫困农村人口向城镇转移。创建产城融合示范区，加快右江河谷城乡一体化进程，吸引农村人口转移。到2020年，新增城镇人口60万人以上。推进城乡一体化与美丽乡村建设结合。

（五）全面推进改革创新，奋力激活发展新动力。

全面深化改革。推进供给侧结构性改革，抓好去产能、去库存、去杠杆、降成本、补短板五大任务。继续取消和下放一批行政权限。推进百色氮肥厂、百色通用机械厂等企业改制，加快右江水务公司挂牌上市，组建百色旅游文化投资公司。稳步推进混合所有制经济。大力支持非公经济发展。深化投融资体制改革，完善政银企社合作对接机制，推广政府和社会资本合作新模式。深化财税体制改革，建立现代财政制度。推动农村土地、集体产权、农业经营模式、城乡一体化等方面改革取得新突破。深化医药卫生体制综合改革。加快社会诚信体系建设。推进科技、教育、文化、体育、社会保障、生态文明等领域改革，抓好沿边金融、新型城镇化等全国全区改革试点工作。

大力推进创新驱动。创建百色国家农业高新技术产业开发区。推动重点领域关键技术突破，在生态铝、冶金、机械、石化、建材、食品、农产品加工等取得新成果。加强科技创新平台建设，推动产学研合作，做大做强国家铝金属产品检验中心，建立自治区级工程技术研究中心、产业技术创新战略联盟、自治区高新技术企业、自治区级创新型企业各15家以上。实施知识产权创新工程，培育一批知识产权示范企业。实施“质量兴市”战略，加强品牌建设，力争获得广西名牌产品30个以上、广西著名商标70件以上，实现全国知名品牌示范区创建、主席质量奖零的突破。推进大众创业万众创新。引进培养高层次人才和紧缺人才，壮大创新人才队伍。

（六）融入“一带一路”战略，奋力打造开放发展新格局。

加快沿边开放开发。加强与越南对接，完善区域基础设施互联互通。推动龙邦口岸升格为国际口岸，实现龙邦进境水果指定口岸通过考核验收并运营。推进平孟、岳圩口岸提级升格。筹划建设那坡弄平国家一类口岸、百色水运口岸，筹划设立百色海关。加快推进中国龙邦—越南茶岭跨境经济合作区、百色（靖西）边境经济合作区、平孟综合物流园区、中国—柬埔寨合作产业园、田东锦江集团与印尼西加里曼丹省合作产业园、百色（德保）进出口加工园区、百色保税物流中心建设，创建中国—东盟农产品自由贸易示范区、沿边开发开放创新示范区，打造面向东盟的重要开放门户城市。

拓展国内区域合作。加快建设百色—文山、百色—黔西南跨省经济合作园区，推进滇桂黔石漠化片区治理。加强与重庆、成都、西安等地区在现代制造业、商贸物流、信息技术对接合作，融入丝绸之路经济带。强化区内合作，推进左右江革命老区振兴发展。参与北部湾经济区开放开发。对接桂台、泛珠三角、长三角，加强在投融资、人才交流、教育科技、劳务输出等领域和产业转移合作。力争到2020年全市区外招商引资到位资金累计达2100亿元以上。

大力发展对外贸易。扩大铝、锰、碳素、农机、建材、茶叶等优势产品出口，积极培育中医药、电子信息、机电等产品贸易增长点。实施加工贸易倍增计划，引进和培育加工贸易龙头企业，发展机械、电子、新材料、轻工业品等加工贸易。引进企业参与边境贸易。设立对外贸易发展促进基金，培育一批外贸骨干龙头企业。力争到2020年外贸进出口总额突破100亿美元。

（七）坚持绿色发展，奋力建设生态文明城市。

夯实生态基础。推进石漠化治理、退耕还林、珠江防护林、生态公益林、绿色矿山建设等生态治理工程。抓好岑王老山、金钟山、雅长林场、福禄河、浩坤湖、芦仙湖等国家级自然保护区和湿地公园建设管理。构建珠江上游重要生态屏障。加强澄碧河水库、天生桥等库区生态环境保护。落实生态补偿机制，探索市场化生态补偿有效方式。创建“国家森林城市”和“国家生态文明示范市”，稳步推进生态乡村、宜居乡村、幸福乡村建设。

扎实抓好节能减排。优化能源结构，加快淘汰落后产能。加强高耗能行业监管，推行合同能源管理。严格控制碳排放。对重点行业重点区域总氮、总磷和挥发性有机物实施总量控制。对能源和水资源消耗、建设用地实行总量和强度双控制。建立重点排污单位清单，全面推行排污许可证管理制度，探索建立排污权交易试点。加大建筑节能改造力度，推动绿色建筑发展。

加强环境保护和治理。建立实时在线环境监控系统和在线监测体系。严格实施水、大气、土壤污染防治三大行动计划。加快推进重点流域水环境综合治理。加大工业废水处理设施建设，严格控制工业污水排放。狠抓工业废气、机动车尾气和城市建设工地扬尘整治。推广科学施肥，遏制土壤污染。加强社会噪声、工业噪声和生活噪声管控。加强农业农村环境污染防治。

大力发展生态经济。加强对铝工业、石化、制糖等资源型工业进行生态化改造。加大矿产资源循环利用和赤泥尾矿的综合利用。建设生态有机农业示范基地，培育发展特色有机果蔬、有机茶叶、有机水稻。推广标准化畜禽生态养殖业、现代生态渔业，促进农业生产向绿色有机循环发展。支持凌云县等县（市、区）创建全国有机农业示范县。鼓励重大环境治理领域引入第三方参与，推广绿色产品和绿色服务，扩大生态服务业发展规模。做好“三废”综合利用，建设再生铝资源产业园区。

（八）补齐短板，奋力实现发展成果由人民共享。

扩大就业和优化分配。实施更加积极的就业政策，注重创业带动就业，全市城镇新增就业10万人以上，农村劳动力转移就业24万人次以上。建立和完善劳动者自主择业、市场调节就业和政府促进就业相结合的机制，努力促进充分就业。建立收入稳定增长机制，努力提高居民收入水平。完善收入分配制度，规范收入分配秩序，建立和谐的劳动关系。

健全社会保障体系。深入实施全民参保计划。健全公平统一规范的城乡居民养老保险机制，建立健全机关事业单位工作人员养老保险制度。整合城镇居民基本医疗保险和新型农村合作医疗保险制度，建立城乡统一的居民基本医疗保险制度。推进城乡居民大病医疗保险。健全失业、工伤、生育保险制度。扩大城镇保障性住房有效供给，提高住房保障水平。扩大社会救助覆盖面，提高社会救助政策兜底脱贫水平，发展社会福利、慈善事业和残疾人事业。支持和鼓励社会工作服务机构发展。建立健全留守儿童、留守妇女、留守老人和残疾人关爱服务体系。

全面提升教育水平。加快发展学前教育，鼓励建设公建民办、民建民办幼儿园，大力发展普惠性幼儿园，到2020年，学前三年毛入园率达85%以上。推进义务教育“全面改薄”工程，加快标准化学校建设，逐步消除“大班额”、“大通铺”现象，到2020年，九年义务教育巩固率达95%。加快普及高中阶段教育。全面推进免费中等职业教育，组建百色职业教育集团，建设职业教育园区。创建百色大学园区，办好平果大学园。大力支持民办教育发展。努力办好特殊教育。畅通继续教育、终身教育通道，加快教育信息化建设，发展远程教育。加大教师队伍培养和引进力度。

推进健康百色建设。健全县乡村三级医疗卫生服务网络。优化中心城区医疗卫生机构布局。加强卫生计生综合监督执法体系建设和医疗质量监管。加强乡村医生队伍建设。积极发展民族医药。加强禁毒与艾滋病防治。完善食品药品安全监管制度，建立健全食品药品安全治理体系。创建农产品安全示范县、食品安全示范城市。优化计生服务管理，完善人口计生目标管理考核机制，巩固计划生育优质服务成果。发展医养融合养老服务。

全面加强文化建设。大力传承和发展民族文化，推进国家级百色壮族文化生态保护区规划建设，创建历史文化名城和民族文化名市。实施文化建设八大工程，加强文化体育基础设施建设，加快文化体育产业发展，推进百色红色文化产业园、养生文化产业园、特色体育基地等重大项目建设。增加农村群众性公共文化体育产品和服务。推进建设一批妇女、儿童、青少年文体活动场所。加强社会主义核心价值观教育，大力弘扬百色起义精神，全面提高社会文明素质。

推进平安百色建设。加强和创新社会治理，全面提升社会治理效能，构建全民共建共享的社会治理新格局。严厉打击各种违法犯罪活动，维护社会大局稳定。强化安全生产“党政同责、一岗双责、失职追责”，落实属地责任和行业监管责任，全面实施“五安工程”。加强自然灾害监测预警，提高突发事件应急处置能力。

大力支持部队和国防建设，加强国防动员和后备力量建设，推进军民融合和双拥工作，巩固和发展军政军民团结。加强和改进新形势下民族工作，不断推进各民族团结进步繁荣。充分发挥工会、共青团、妇联、残联、红十字会、科协、文联等群团组织作用。继续做好宗教、外事、侨务、民族、新闻出版、水库移民、统计、国资监管、粮食、供销、气象、地震、测绘、人事、编制、档案、老龄、地方志、决策咨询、哲学社会科学等工作。

三、全力实现“十三五”良好开局

今年以来，全市经济发展呈现总体平稳、稳中向好、逆势上扬势头。上半年，全市地区生产总值增长9%，排全区第2位；财政收入增长14.3%，排全区第1位；规模以上工业总产值增长17.7%，排全区第1位；规模以上工业增加值完成224.8亿元，增长9.6%；固定资产投资完成504.7亿元，增长16.2%；社会消费品零售总额完成115.4亿元，增长10.4%；城镇居民人均可支配收入13909元，增长8%；农村居民人均可支配收入4482元，增长11.1%。为“十三五”开好局夯实了基础。后面3个月，我们将坚持目标不变、任务不减、力度不降，克难攻坚，奋力实现地区生产总值增长10%以上，确保各项目标任务全面完成。

（一）全力抓好投资和项目建设。着力推进交通、工业、城建、农林水、科教文卫等重点项目建设。抓好百色至河池、靖西至龙邦、百色至乐业高速公路项目建设，建成云桂高铁百色至昆明段。推进巴马至平果坡造、巴马燕洞至田东、百色至泮水等国省公路干线改扩建工程。加快百东新区路网及配套设施工程建设，建成百色干部学院。实施好城区功能提升“十大工程”等重大项目。促进民间投资稳步回升。确保年度固定资产投资完成1150亿元，增长12.5%。

（二）全力推进工业复产达产超产。

完善区域电网一期工程基础建设，推动百矿、银海铝、信发铝等企业复产、达产、超产，实现区域电网新增负荷135万千瓦，带动新增电解铝25万吨、碳素13万吨、铝深加工20万吨。继续落实自治区降成本“41条”和我市降成本“43条”，着力解决企业生产用电用气成本高、融资难、用矿难等问题，促进一批企业稳定运行，实现一批企业新建成投产，培育一批规模以上工业企业。确保年度规模以上工业增加值完成473亿元，增长12.5%。

（三）全力保持农业增速居全区前列。抓好秋冬农业生产，加强沙糖桔、柑橙和秋冬菜等果蔬的生产销售。完成种植业新增2.5亿元、养殖业新增1.4亿元、林业新增1亿元目标。办好第九届中国—东盟现代农业展示交易会、农产品展销会、推介会等农产品推介活动。力争第一产业实现174亿元，增长4.8%。

（四）全力促进消费出口稳增长。培育旅游、汽车、住房、电商等消费热点。加快推动视虎呼叫中心、云计算产业园等一批新业态重点项目正式运营投产。推进万生隆国际商贸物流中心一期工程和岳圩、新兴边民互市点开放运营，落实边小贸易扶持政策，促进外贸进出口稳定回升，力争全年外贸进出口总额完成19.7亿美元，增长20%。

（五）全力抓好财税金融工作。加强财政收入组织工作，确保实现增长7%的目标任务。继续清理财政存量资金，加快财政支出进度。加快推进全国政策性金融扶贫实验示范区建设，争取农业发展银行追加扶贫专项贷款额度，加大信贷投放。加快左右江革命老区振兴发展基金、百色城镇化发展基金、百色扶贫开发基金等融资平台建设。

（六）全力完成年度脱贫摘帽任务。扎实推进“八个一批”、“十大行动”和“十个到村到户”。完善“双承诺、双认定、双确认”工作机制，强化脱贫攻坚责任落实和绩效考核。确保实现右江区、田东县2个贫困县（区）摘帽，力争田阳县提前脱贫摘帽。确保全市112个贫困村出列，20万贫困人口脱贫，力争新增137个村出列、3.6万人脱贫。加快推进与深圳市的东西部扶贫协作。

（七）全力抓好环保和安全生产。抓好中央环境保护督察反馈问题的整改。强化城区扬尘治理。严格节能减排，确保万元生产总值能耗下降3%，主要污染物排放量控制在自治区下达目标内。推进城镇污水管网建设及消灭污水直排口工作。完成植树造林50万亩以上。强化矿山、尾矿库、危化品、建筑施工、交通运输、旅游等重点行业领域安全防范。加强食品药品安全监管。

（八）全力保障民生目标实现。确保全年城镇居民人均可支配收入增长8.5%，农村居民人均纯收入增长10%。五项社会保险参保人数达127.2万人次。城镇新增就业2万人以上，农村劳动力转移就业新增4.8万人次以上。全面完成为民办实事工程。

做好今后五年各项工作，必须进一步加强政府自身建设。不断提高新常态下推动改革发展的能力和水平，提高政府执行力。进一步改进作风，提高政府工作效率。深入开展“七五”普法。做好地方性法规和政府规章立法工作，规范行政权力，强化严格执法，推进依法行政，基本建成法治政府。自觉接受人大及其常委会的法律监督、工作监督，积极支持人民政协履行政治协商、民主监督、参政议政职能，切实做好人大代表建议和政协提案办理工作。认真听取各民主党派、工商联、人民团体和社会各界人士的意见。健全依法决策机制，充分发挥监察、审计等专门监督的作用，高度重视人民群众监督和新闻舆论监督，及时回应社会关切，提高政府公信力。推行政府部门权力清单和责任清单公开制度，全面推进政务公开。坚持不懈推进党风廉政建设和反腐败工作。努力建设为民、务实、创新、高效、清廉的服务型政府。
